# Supplementary figures and images for: ORION software tool for the geometrical calibration of all-sky cameras
Source: PLoS One. 2022 Mar 31;17(3):e0265959. doi: 10.1371/journal.pone.0265959 (PMC8970509; doi:10.1371/journal.pone.0265959)

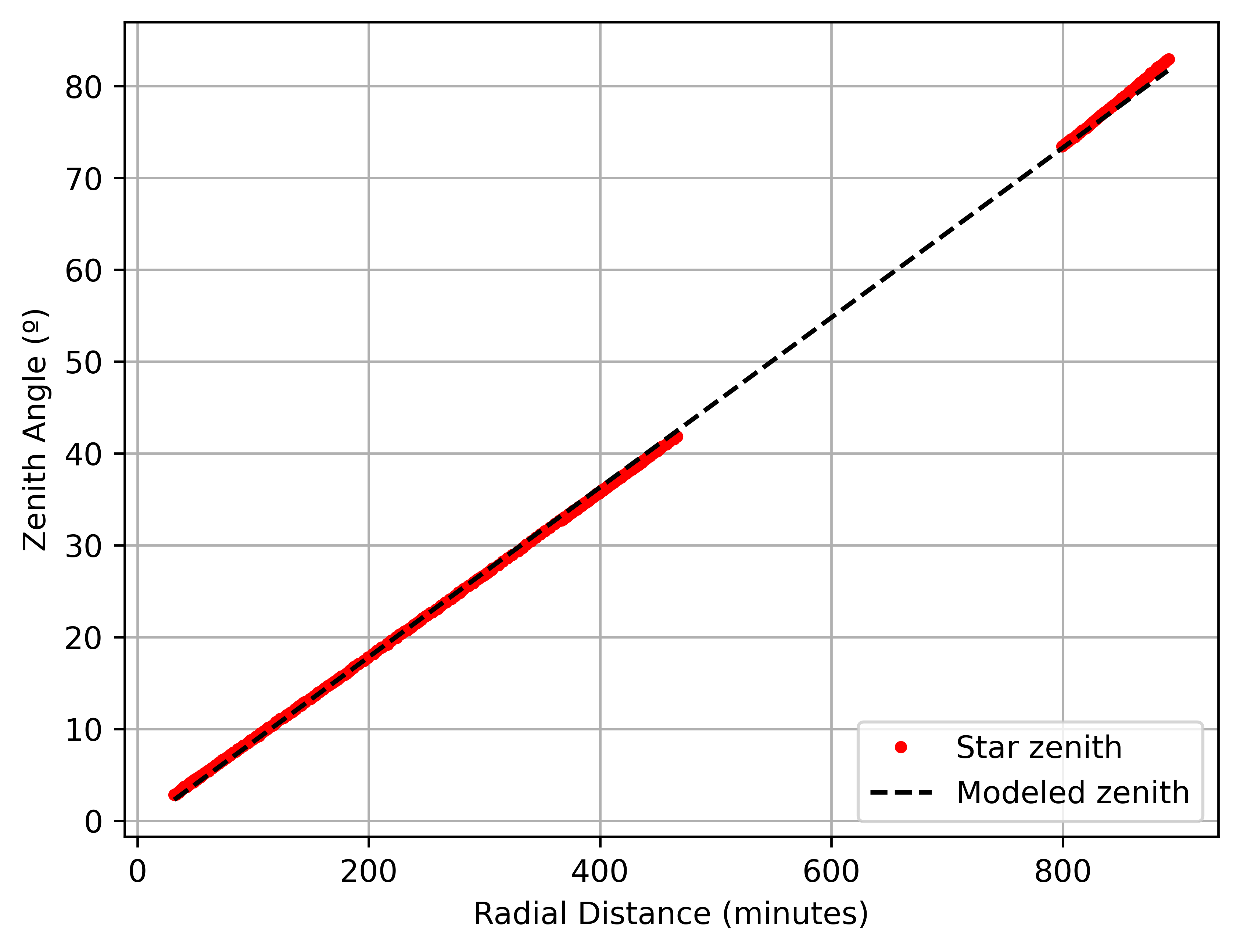

Supplement: S1 Fig — (TIFF) [file pone.0265959.s001.tiff]
