# Supplementary material for: ORION software tool for the geometrical calibration of all-sky cameras
Source: PLoS One. 2022 Mar 31;17(3):e0265959. doi: 10.1371/journal.pone.0265959 (PMC8970509; doi:10.1371/journal.pone.0265959)
Supplement: Attachment — Submitted filename: PONE-D-21-34079_annotated.pdf [file pone.0265959.s002.pdf]

PLOS ONE

ORION software tool for the geometrical calibration of all-sky cameras

--Manuscript Draft--

**Manuscript Number:**	PONE-D-21-34079
**Article Type:**	Research Article
**Full Title:**	ORION software tool for the geometrical calibration of all-sky cameras
**Short Title:**	ORION software tool
**Corresponding Author:**	Juan Carlos Antuña-Sánchez, MSc.<br>Universidad de Valladolid<br>Valladolid, Valladolid SPAIN
**Keywords:**	all-sky camera, calibration, stars, sky,
**Abstract:**	This paper presents the software application ORION (All-sky camera geOmetrycalibRation from star positIONs). This software has been developed with the aim ofproviding geometrical calibration to all-sky cameras, to know what sky coordinates(zenith and azimuth angles) are seen by each camera pixel. It is useful to locate bodiesover the celestial vault, like stars and planets, in the camera images. To obtain thecalibration matrices, the user needs to feed ORION with a set of cloud-free sky imagescaptured at night-time. ORION looks for the position of many stars in the sky images.This search can be automatic or manual. The sky coordinates of the stars and thecorresponding pixel positions in the camera images are used together to determine thecalibration matrices by three factors: the pixel position of the sky zenith in the skyimage; the shift angle of the azimuth viewed by the camera with respect to the realNorth; and the relationship between the sky zenith angle and the pixel radial distanceregarding the sky center in the image. In addition, ORION includes other features tofacilitate its use by the user, such as exporting data to different formats, checking theaccuracy of the calibration or calculating the field of view of each pixel. An exampleabout the use of ORION is shown, obtaining the calibration matrices for a set of imagesand studying the accuracy of the calibration to predict a star position. This accuracy isabout 9.0 arcmin in average for an analyzed example using a camera with a 5.4arcmin/pixel resolution (accuracy about 1.7 pixels).
**Order of Authors:**	Juan Carlos Antuña-Sánchez, MSc.
	Roberto Román, PhD.
	Juan Luis Bosch, PhD.
	Carlos Toledano, PhD.
	David Mateos, PhD.
	Ramiro González, PhD.
	Victoria Cachorro, Proffesor
	Ángel de Frutos, Proffesor
**Additional Information:**	
**Question**	**Response**
**Financial Disclosure**<br><br>Enter a financial disclosure statement that describes the sources of funding for the work included in this submission. Review the submission guidelines for detailed requirements. View published research articles from *PLOS ONE* for specific examples.	No, The funders had no role in study design, data collection and analysis, decision to publish, or preparation of the manuscript.

Powered by Editorial Manager® and ProduXion Manager® from Aries Systems Corporation

This statement is required for submission and **will appear in the published article** if the submission is accepted. Please make sure it is accurate.<br><br>**Unfunded studies**<br>Enter: *The author(s) received no specific funding for this work.*<br><br>**Funded studies**<br>Enter a statement with the following details:<br>• Initials of the authors who received each award<br>• Grant numbers awarded to each author<br>• The full name of each funder<br>• URL of each funder website<br>• Did the sponsors or funders play any role in the study design, data collection and analysis, decision to publish, or preparation of the manuscript?<br>• **NO** - Include this sentence at the end of your statement: *The funders had no role in study design, data collection and analysis, decision to publish, or preparation of the manuscript.*<br>• **YES** - Specify the role(s) played.<br><br>* typeset	
**Competing Interests**<br><br>Use the instructions below to enter a competing interest statement for this submission. On behalf of all authors, disclose any competing interests that could be perceived to bias this work—acknowledging all financial support and any other relevant financial or non-financial competing interests.<br><br>This statement is required for submission and **will appear in the published article** if the submission is accepted. Please make sure it is accurate and that any funding sources listed in your Funding Information later in the submission form are also declared in your Financial Disclosure statement.<br><br>View published research articles from	The authors have declared that no competing interests exist.

PLOS ONE for specific examples.<br><br>**NO authors have competing interests**<br><br>Enter: *The authors have declared that no competing interests exist.*<br><br>**Authors with competing interests**<br><br>Enter competing interest details beginning with this statement:<br><br>*I have read the journal's policy and the authors of this manuscript have the following competing interests: [insert competing interests here]*<br><br>* typeset	
**Ethics Statement**<br><br>Enter an ethics statement for this submission. This statement is required if the study involved:<br><br>• Human participants<br>• Human specimens or tissue<br>• Vertebrate animals or cephalopods<br>• Vertebrate embryos or tissues<br>• Field research<br><br>Write "N/A" if the submission does not require an ethics statement.<br><br>General guidance is provided below. Consult the submission guidelines for detailed instructions. **Make sure that all information entered here is included in the Methods section of the manuscript.**	N/A

**Format for specific study types**<br><br>**Human Subject Research (involving human participants and/or tissue)**<br>• Give the name of the institutional review board or ethics committee that approved the study<br>• Include the approval number and/or a statement indicating approval of this research<br>• Indicate the form of consent obtained (written/oral) or the reason that consent was not obtained (e.g. the data were analyzed anonymously)<br><br>**Animal Research (involving vertebrate animals, embryos or tissues)**<br>• Provide the name of the Institutional Animal Care and Use Committee (IACUC) or other relevant ethics board that reviewed the study protocol, and indicate whether they approved this research or granted a formal waiver of ethical approval<br>• Include an approval number if one was obtained<br>• If the study involved *non-human primates*, add *additional details* about animal welfare and steps taken to ameliorate suffering<br>• If anesthesia, euthanasia, or any kind of animal sacrifice is part of the study, include briefly which substances and/or methods were applied<br><br>**Field Research**<br><br>Include the following details if this study involves the collection of plant, animal, or other materials from a natural setting:<br>• Field permit number<br>• Name of the institution or relevant body that granted permission	
**Data Availability**<br><br>Authors are required to make all data underlying the findings described fully available, without restriction, and from the time of publication. PLOS allows rare exceptions to address legal and ethical concerns. See the PLOS Data Policy and FAQ for detailed information.	Yes - all data are fully available without restriction

A Data Availability Statement describing where the data can be found is required at submission. Your answers to this question constitute the Data Availability Statement and **will be published in the article**, if accepted.<br><br>**Important:** Stating 'data available on request from the author' is not sufficient. If your data are only available upon request, select 'No' for the first question and explain your exceptional situation in the text box.<br><br>Do the authors confirm that all data underlying the findings described in their manuscript are fully available without restriction?	
**Describe where the data may be found in full sentences. If you are copying our sample text, replace any instances of XXX with the appropriate details.**<br><br>• If the data are **held or will be held in a public repository**, include URLs, accession numbers or DOIs. If this information will only be available after acceptance, indicate this by ticking the box below. For example: *All XXX files are available from the XXX database (accession number(s) XXX, XXX.*).<br>• If the data are all contained **within the manuscript and/or Supporting Information files**, enter the following: *All relevant data are within the manuscript and its Supporting Information files.*<br>• If neither of these applies but you are able to provide **details of access elsewhere**, with or without limitations, please do so. For example:<br><br>*Data cannot be shared publicly because of [XXX]. Data are available from the XXX Institutional Data Access / Ethics Committee (contact via XXX) for researchers who meet the criteria for access to confidential data.*<br><br>*The data underlying the results presented in the study are available from (include the name of the third party*	All source code and example data files are available from the Zenodo (10.5281/zenodo.5595851).

and contact information or URL).<br>• This text is appropriate if the data are owned by a third party and authors do not have permission to share the data.<br><br>* typeset	
Additional data availability information:	Tick here if the URLs/accession numbers/DOIs will be available only after acceptance of the manuscript for publication so that we can ensure their inclusion before publication.

Powered by Editorial Manager® and ProduXion Manager® from Aries Systems Corporation

Manuscript
Click here to access/download;Manuscript;MainText.pdf

ORION software tool for the geometrical calibration of all-sky cameras

J.C. Antuña-Sánchez[1*], R. Román[1], J.L. Bosch[2], C. Toledano[1], D. Mateos[1], R. González[1], V.E. Cachorro[1], A.M. de Frutos[1],

1 Group of Atmospheric Optics, Universidad de Valladolid (GOA-UVa), Valladolid, 47011, Spain
2 Departamento de Química y Física, Universidad de Almería, Almería, 04120, Spain

* jcantuna@goa.uva.es

Abstract

This paper presents the software application ORION (All-sky camera geOmetry calibRation from star positIONs). This software has been developed with the aim of providing geometrical calibration to all-sky cameras, to know what sky coordinates (zenith and azimuth angles) are seen by each camera pixel. It is useful to locate bodies over the celestial vault, like stars and planets, in the camera images. To obtain the calibration matrices, the user needs to feed ORION with a set of cloud-free sky images captured at night-time. ORION looks [illegible]he position of many stars in the sky images. This search can be automatic or manual. The sky coordinates of the stars and the corresponding pixel positions in the camera images are used together to determine the calibration matrices by three factors: the pixel position of the sky zenith in the sky image; the shift angle of the azimuth viewed by the camera with respect to the real North; and the relationship between the sky zenith angle and the pixel radial distance regarding the sky center in the image. In addition, ORION includes other features to facilitate its use by the user, such as exporting data to different formats, checking the accuracy of the calibratio[illegible] calculating the field of view of each pixel. An example about the use of ORION is shown, obtaining the calibration matrices for a set of images and studying the accuracy of the calibration to predict a star position. T[illegible] accuracy is about 9.0 arcmin in average for an analyzed example using a camera with a 5.4 arcmin/pixel resolution (accuracy about 1.7 pixels).

1 Introduction

All-sky cameras are instruments capable of capturing images of the full sky from the ground. There [illegible]many varieties of this type of instrument: with electronic sensors (CMOS or CCD) or film (especially in the past, see [1] and references therein); with monochromatic sensors or with filters, typically RGB Bayer filters but also narrower; looking to a mirror oriented to the sky or looking directly to the sky with a fish-eye lens; static cameras or moving cameras, usually installed on a sun-tracker with a shadow ball to block the direct sun light; operating at daytime, night-time or both; and others. Some of the best features of the current all-sky cameras are: they allow the possibility of changing exposure time, sensor gain and other parameters to adapt the camera to the sky scenario: they are able to obtain a snapshot of the full sky radiance, covering every sky angle and in various spectral ranges; the capture time is short; and they are economic as compared to other instruments. Conversely, obtaining an accurate

radiometric calibration of the images is difficult, the filters are generally too wide for some applications, and there are also issues with the presence of hot pixels, pixel saturation, lens aberrations, and image vignetting, among others.

This kind of instruments is generally used to observe and quantify clouds and cloud cover [2–10] or as a proxy of the sky conditions. However, all-sky cameras present high versatility and they can also be used for different purposes: to derive other more complex cloud properties as cloud base height by stereoscopic methods [11,12]; to detect and observe aurora, celestial bodies or bolides [13–15]; to estimate the sky radiance [16–18]; to study the cloud effects over solar radiation [19,20]; to study the polarization of the sky light [21,22]; to detect and retrieve atmospheric aerosol properties [23–25]; to obtain synergy in combination with other instruments like radiometers, ceilometers or photometers [26–28], among others.

The knowledge of the sky coordinates that are represented in each pixel of an all-sky camera is crucial in several applications; for example to extract sky radiance at specific sky angles [25,28], to forecast solar irradiance [29,30], to calculate aurora and cloud base altitudes by stereographic methods [31,32], or simply to locate any body over the celestial vault. This can be achieved by a geometrical calibration of the all-sky cameras, which ~~basically~~ consists [illegible]btaining two matrices of the same size than the camera images but with the values of the both azimuth and zenith angles viewed by each pixel. ~~For this purpose~~, several calibration methods have been described in the literature[illegible] Intrinsic calibratio[illegible]r determining the internal parameters of the camera~~, is usually~~ [illegible] recording images of a chessboard, such a[illegible]amCalib toolbox [33,34]~~, w~~here the corners of the chessboard are identified and used to detect any distortion on the camera optics. Extrinsic camera calibrations, consisting of the determination of the camera orientation in the local reference frame [30], are generally based on the identification of the positions of the Sun [32,35,36] or any star [28,37,38] in several images and on the correlation of these positions with the Sun or star coordinates.

~~In this framework,~~ we have developed the software application named ORION (all-sky camera geOmetry calibRation from star positIONs), with the main objective of providing an open and free tool for the geometrical calibration of all-sky cameras that is accurate, simple and user-friendly.The use of stars instead of the Sun for the geometric calibration is chosen because the Sun size in camera images is usually larger (problems to identify its center in the image) and, in addition, by using multiple stars we are able to cover more sky angles. ORION is written in python3 language and Qt5, and it is capable of geometrically calibrating an all-sky camera by identifying star positions in the celestial vault. The source code and example data are hosted at Zenodo (`https://doi.org/10.5281/zenodo.5595851`). The Windows build of the application is hosted on the GOA-UVa website (`http://goa.uva.es/orion-app/`).

This paper introduces the ORION application and it is structured as follows: Section 2 introduces the used instrumentation, the workflow and the theoretical principles behind ORION while 3 presents an example of the use of ORION. Finally, the main conclusions are summarized in Section 4.

2 Instrumentation, data and method

2.1 All-sky camera

In order to show how ORION works, sky images from an all-sky camera have been used. This camera is installed at the scientific platform of the GOA-UVa (Atmospheric Optics Group, University of Valladolid) located on the rooftop of the Faculty of Sciences of Valladolid (Spain; 41.66360° N, 4.70583° W, 705 m a.s.l.). More information about the GOA-UVa platform and the climatic conditions of Valladolid can be found in [18,39–41].

The all-sky camera model is an "OMEA 3C" from *Alcor System* manufacturer. It is formed by a sensor with a fisheye lens, both encapsulated in a weatherproof case with a BK7 glass dome on top. The case includes a heating system to avoid water condensation on the dome. The camera sensor is a SONY IMX178 RGB CMOS sensor, with an image size of 3096 X 2080 pixels, a pixel scale of 5.4 arcmin/pixel and 14-bit resolution.

This all-sky camera is configured to capture a multi-exposure set of raw sky images every 2 minutes at night-time and every 5 minutes at daytime. These raw images are stored and then properly converted to 8-bits true color or gray-scale images. The gray-scale images are used to carry out the geometrical calibration. A set of these sky images captured at 23rd August 2020 from 20:20 UTC to 23:58 UTC (110 images) is used for the example shown in Section 3.1

2.2 Identification of stars

The data necessary for geometrical calibration purposes are just the position (x and y pixel coordinates) of the center of any star and the sky coordinates of the chosen star (azimuth and zenith angles).To calculate these azimuth and zenith values of a chosen star, the position of the observer (all-sky camera) is required, which is determined by the latitude, longitude, and elevation above sea level of the all-sky camera. ORION obtains the star coordinates for each image from "pyephem" library [42], using as input the mentioned all-sky camera coordinates and the date and time when the image was captured. If the zenith angle of a star is above 83°in an image, ORION does not consider this star for calibration in [illegible]image since it is close to the horizon, whe[illegible]ar identification i[illegible]re difficult due to city lights.

[illegible]nerally, a set of several images obtained at different times is used in the geometrical calibration to cover a wider range of pixel positions and angles. To this end, the ORION user must prepare and put this [illegible]n a folder and introduce the folder path in ORION[illegible]ION reads the date and time of each image directly from the image filename, hence the user must prepare the image set ta[illegible]g into account that date and time of each image must be included in the filename with the following format: "text_YearMonthDay_HourMinute" (example: C006_20200817_0300.jpg). ORION includes an additional utility that allows changing the filename format of the images (see Section 2.4).

Once a star is chosen, its coordinates are obtained as explained. After that, ORION only needs to identify the position of the center of the chosen star in each sky image. The flowchart of this ORION task is described in Figure 1. Each analyzed image is firstly converted into gray-scale. Then, the position of the chosen star is directly determined by ORION as the brightest pixel in a specific area of the image which we named "Region Of Interest" (ROI). Two different ways to do that are implemented in ORION: Manual and Automatic mode, and the ROI depends on the selected mode.

Fig 1. Flowchart for star selection modes.

The Manual mode allows the user to manually choose the ROI. for each sky image, ORION opens an additional window showing the sky image where the user must select the ROI as a rectangular pixel box. For this, ORION uses the function selectROI that is native part of OpenCV library [43]. In order to know where the ROI must be manually selected, or to check if ORION identifies the stars positions correctly, either in Manual or Automatic mode, we recommend the use of Stellarium (`https://stellarium.org`), which is a free open source planetarium showing a realistic hemispherical sky similar to the one captured by a fisheye lens [44].

Automatic mode skips the ROI selection image by image, simplifying the process. However, some previous information about the camera calibration is needed. Depending

on this previous knowledge, ORION allows two options for this mode: Custom and Default. Both options are based on the use of calibration matrices. ORION uses these matrices to find the position of the pixel closest to the chosen star; Harvesine distance function is used for this calculation [45]. If the accuracy of the previous calibration matrices is admissible, the real image of the star in the sky image must be close to the predicted pixel, although not necessarily in the same pixel. Hence, ORION looks for the brightest pixel within a box centred in the pixel predicted by calibration matrices. The size of the mentioned box depends on the size of the full image and also on the chosen option (Custom or Default). ORION assumes that the center of the chosen star is located in the image in its brightest pixel and it stores the x and y position of that pixel.

Custom option uses calibration matrices that were obtained in a previous calibration, for example using Manual mode. In this case, the box used to find the star position is a square box which side dimension is the height (or the width if it is higher) of the sky image divided by 100. This arbitrary value was chosen after running various tests and seeing empirically that it worked best for different images.

Default option is similar to the Custom one, but the calibration matrices are generated from two input parameters instead of the matrices obtained from a previous calibration. These input parameters are: 1) the azimuth shift from North, which indicates the angle between the North of the image (assumed as the top center of the image) and the real geographical North observed in the image; and 2) the extreme zenith, which is the sky zenith angle viewed by the pixel located in the top row and center column in the image. With this information, and assuming that the center of the entire sky (zenith) corresponds to the center of the image, a calibration azimuth and zenith matrices can be calculated. Figure 2 shows a couple of examples of the matrices generated by this method in a 2000x2000 pixel image. The first case (Figs. 2a and b) presents no shift from North, being the azimuth from 0°to 360°in counterclockwise, and a high range of zenith angles; while the second case (Figs. 2c and d) presents a well defined shift from North of -50°and a lower variation in zenith angles due to the lower value of the extreme zenith angle. Finally, the square box used to find the stars in Default option is similar to that in Custom mode, but dividing by 50 instead of 100 (i.e. larger box), since the method of Default option is less accurate.

Fig 2. Defaults matrices for azimuth and zenith . a) Shift from north 0°. b) Extreme zenith 100°. c) Shift from north -50°. d) Extreme zenith 80°.

Once the pixel position of a star in one image is obtained, the user must decide if ORION identifies this position correctly. If it was done correctly, ORION stores the x and y position of the star in the image t[illegible]her with the real star azimuth and zenith angles. If there are more images where identify the same star, ORION continues with the next one until the entire list is finished. The obtained array is stored in a *.npy* file (Numpy file). This mode is useful to perform a quick calibration as an input to the automatic star detection mode. More files can be generated for other stars.

2.3 Calibration algorithm

The position of a pixel in an image is given in Cartesian coordinates, where x and y coordinates are the column and row numbers, respectively, and the system is centred in the left-top pixel (x=0, y=0). The first element corresponds to zero position for x and y because ORION is programmed in python3 language. It is convenient to convert this system into polar coordinates, which is more similar to t[illegible]bserved in the sky by zenith and azimuth angles. In this sense, a polar system centred in the zenith of the sky with Cartesian coordinates equal to x=x_C and y=y_C, can be described as:

$$r = \sqrt{(x_C - x)^2 + (y_C - y)^2} \quad (1)$$

$$\Phi = \arctan\left(\frac{x_C - x}{y_C - y}\right) \quad (2)$$

where r is the radial distance in pixels from the center, and Φ the polar angle in this new system. This polar angle presents its zero value in the direction of y-axis in a similar way t[illegible] in Fig. 2a.

Radial distance and polar angle are directly related to the sky zenith and azimuth angles, respectively. Assuming polar symmetry and that the camera is well levelled, the polar angle must be equal to the sky azimuth but with a shift due to a non perfect alignment of the camera with respect to the North. This shift is the same used in the "Default" option of Automatic mode in Section 3.1. Sky zenith angle is related to radial distance, but this relationship can be linear (which is assumed in the mentioned "Default" option), or a higher polynomial degree. Hence, if we determined the x_C and y_C coordinates, we will obtain the polar coordinates of Eq. 1 and 2; then, if we determine the shift of polar angle with respect to the sky azimuth, and the relationship between radial distance and zenith angle, we can transform the obtained polar coordinates into the sky angles viewed by each pixel. This is the way that ORION uses to calculate the calibration matrices.

The required x_C and y_C values, the azimuth shift from polar coordinates, and the relationship between zenith angle and radial distance can be obtained from the stored data in the previous Section 2.2: real azimuth and zenith star values and x and y pixel positions where the stars were found.

First, ORION calculates the x_C and y_C values. It is an iterative process of four iterations, which [illegible]culates the center position with more precision in each iteration. Each iteration consists of scanning different pixel coordinates and assuming that they are the x_C and y_C values. The first iteration assumes that x_C and y_C values must be near to the center of the image itself (x_{ci}, y_{ci}): x_{ci} = (width-1)/2 and y_{ci} = (height-1)/2. A scan from x = x_{ci} -250 to x= x_{ci}+250 in 5 pixel steps is done. In each scan in x-column an additional scan in y-raw is done from x = y_{ci} -250 to x= y_{ci}+250 in 5 pixel steps too. It implies that ORION tests 101x101 positions (10201) in this iteration for the x_C and y_C values. For each one of these 10201 potential centers, ORION calculates the Φ values by Eq. 2 for all stars positions chosen and previously stored, and then calculates the difference between these Φ values and the real azimuth of the stars (this information was also stored). If the center of the image is well determined, these differences must be constant and equal to the mentioned shift angle. Then, ORION calculates the standard deviation of these differences, and the x and y position of the 10201 tested as center showing the lowest standard deviation is assumed as the true x_C and y_C values.

This first iteration provides a good approximation of the real x_C and y_C values; however, the precision can still be improved, since the scans were done every 5 pixels in order to encompass a big part of the image but expending low computing time. Three more iterations are done by assuming the result of the previous iteration as the initial conditions. The second iteration scans from -50 to 50 around x_{ci} and y_{ci} in 1 pixel step; while the third and fourth scan from -0.5 to 0.5 and from -0.005 to 0.005 in 0.01 and 0.001 pixel steps, respectively. After the four iterations, ORION stops and provides the x_C and y_C values with a precision of 0.001 pixels.

Once x_C and y_C are calculated, the azimuth shift is calculated by ORION using the stored star information. A linear fit between the polar angle obtained using the calculated x_C and y_C and the real azimuth of the stars in the sky is performed by ORION. The y-intercept is considered the azimuth shift angle. This linear fit is done

excluding star positions with zenith angles below 20°, in order to remove pixels for which a little variation in x and y position implies a big variation in the polar angle.

Once azimuth shift and x_C and y_C values are calculated, ORION calculates the radial distance using the stored star information and Eq. 1. A polynomial fit between the stored real zenith angle of the stars and the obtained radial distance of the image is performed. The degree of this polynomial fit is chosen by the user. After that, the zenith angle viewed by each pixel can be directly obtained by applying the fit coefficients to the known radial distance, which is also available from the previously calculated x_C and y_C values. This information is enough to obtain the geometrical calibration matrices of sky azimuth and zenith angles.

2.4 Others features

ORION presents additional utilities to check calibration, rename images, configure default camera data, and convert *.npy* to others formats. These are located in the Tools menu.

Calibration check is performed for a selected star. A *.txt* file can be saved with the data generated from the calibration check. These data are: image date, azimuth, zenith and distance between the selected calibration pixel and the brightest pixel.

ORION requires the image names to be in the format "text_YearMonthDay_HourMinute" (example: C006_20200817_0300.jpg). To facilitate file renaming, the user can select various formats, set the time zone and flip the images horizontally, vertically or both.

The *.npy* files can be converted into Matlab (*.mat*), HDF5 (*.h5*) and csv formats using comma or tab as separator. The input variables latitude, longitude, elevation, image height and width, image path and the path for matrices can be set by default. These can be loaded by clicking on Load default inputs, located in the Tools menu.

3 Results

3.1 Star position detection and calculation of azimuth and zenith matrices

An example of ORION calibration with real images is shown in this section. The images corresponds to the all-sky camera, described in Section 2.1, installed at Valladolid. We selected the images every 2 minutes from 20:20 UTC to 23:58 UTC at 23rd August 2020. We chose this set of images since it corresponds to a clear night with fully cloudless conditions and without Moon presence. First of all, the camera location information and the path for images local folder are introduced in the input parameters. After that we can choose between manual or automatic mode (see Fig 1). Automatic default option is selected since we have no previous custom calibration. The information about default matrices in this example is: the shift from north equal to -5°and extreme zenith equal to 95°. Both parameters are introduced in the application as can be observed in the screenshot of Fig 3.

Fig 3. ORION screenshot of the star identification process in automatic mode under default matrices option. The chosen star is Capella.

Once this previous information is introduced, we start to identify and detect star positions in the sky images. The first chosen star in this example is Capella; this is selected from the list of available stars and the path where the file with the data will be stored is selected. The procedure described here is the same for every star. When the

"Start" button is pressed (see Fig 3), ORION reads, from "pyephem" library, the azimuth and zenith angles corresponding to the time and place of the first image; then, the position of the closest pixel to the star coordinates is obtained following the calibration matrices given by the "Default" input parameters. The position of the brightest pixel inside a square box centred in the mentioned "closest pixel" is considered the position of the center of the chosen star in the image. The size of this square box is the width (or height, whatever is larger) of the image divided by 50. ORION shows the chosen star position marked with a white circle in the main sky image (left side of the application; Fig 3) but also in a zoomed image at the bottom-right corner of the application ("Region of image" in Fig 3). This zoomed image is useful to discern whether the chosen star position is correct or not (hot pixels can lead to mistaken identification). If the user considers that the star position is well identified, the user will click on the "Add point" button and then the pixel position will be ~~ho[illegible]n~~ in the main sky image with a green circle; if not, the user will click on "Remove point" and the pixel will be hold on with a red circle and the star position for this image will not be stored.

Another way to obtain the star position in the image is the "Manual" mode, as it is shown in Fig 4. This method is similar to the automatic one but for each image an additional window ("Select star" in Fig 4) is open showing the sky image to analyze. Then the user manually chooses the box where the position of the brightest pixel is found (see white box with cross inside in Fig 4).

Fig 4. ORION screenshot of identify star process in manual mode.

When a star position is added or removed (either in Manual or Automatic mode), then ORION analyzes the next sky image (see Fig. 1) and repeats the process until all the available images are analyzed or until we click on the "Finish" button. Once it is finished, ORION stores in the chosen file (in this case "capella.npy" in "stars_coordinates" folder) the azimuth and zenith angles of the star and the x and y pixel positions assigned to this star in each sky image added by "Add point" option. This file contains the needed information for the calibration matrix calculation; however, only one star in a night usually does not cover a full variety of zenith and azimuth angles. Therefore, it is recommended to make the calibration with the positions of several stars positions.

In the present example, we repeat the process to obtain the positions of Capella, Altair, Vega and Deneb. Once the four files are generated using the "Default" mode, they are used to obtain the azimuth and zenith calibration matrices of the camera. For this, the "calculate center and calibration matrices" tab in the application is selected. In this tab we introduce the path of the saved files with star and camera pixel positions ("Input files path" in Fig. 5); in this case the four mentioned files for each star (Capella, Altair, Vega and Deneb). This tab also requests a path to save the calibration matrices which will be calculated from the input files. After pressing the "Calculate" button, the software calculates the center of the image and the relationship between the zenith angle and radial distance, as explained in Section 2.3. The degree of the polynomial fit between zenith angle and radial distance can be chosen, being equal to 2 in our example (see right side of Fig. 5), since the degree 1 option (linear fit) does not perfectly agree with the data under high zenith angle values (see Figure S01 in supplementary material). The azimuth and zenith calibration matrices are also calculated from this information and they are shown in the application, as can be observed in the left side of Fig. 5.

Fig 5. ORION screenshot for calculate center and calibration matrices using the stars: Altair, Capella, Deneb and Vega.

The obtained calibration matrices in this example could be used; however, the graph

of Fig. 5 shows that they were calculated without taking into account zenith angles between 40 and 70°. It is recommended to cover the maximum zenith angle range for the calibration, hence, in this example, we will look for more star positions in order to fill the gap of zenith angles. Two additional stars are selected for this purpose: Arcturus and Alphecca.

The pixel positions of Arcturus and Alphecca are retrieved using ”Automatic” mode but ”Custom” option, since a previous calibration is available (Fig. 5), and this option must be more accurate than the ”Default”. This mode is equal to ”Default” but using a pre-calculated azimuth and zenith matrices (the path is introduced as input, see Fig. 6) and considering a smaller square box (100 times smaller than the maximum dimension of the analyzed sky image, see Section 2.2) for finding the brightest pixel. Figure 6 presents an screenshot after the calculation of pixel and star positions for Alphecca is finished. In this case, some pixels have not been properly identified by ORION (the star was mixed up with a hot pixel), hence we have used the ”Remove point” option for them; they can be seen in red color in Fig. 6, indicating that these positions (red circles) are not stored in the ”alphecca.npy” file.

Fig 6. ORION screenshot of star identification process in automatic mode under custom matrices option. The chosen star is Alphecca.

In this example, the calibration matrices are recalculated adding the information of these two new stars in the ”Calculation center and calibration matrices” tab as shown in Fig. 7. Now the fitting between zenith angles and radial distance is done with a complete zenith angle range, and the second order polynomial relationship can be better observed. As a final result, the center of the sky image, which corresponds to the sky zenith, is really close to the center of the sky image: pixel (1000, 1000). Regarding the shift from North, it is -5.23°, which is also close to our a priori approach of -5.0°when we used ”Default” option in ”Automatic” mode.

Fig 7. ORION screenshot for calculating center and calibration matrices using the stars: Alphecca, Altair, Arcturus, Capella, Deneb and Vega.

3.2 Calibration check

Once the final calibration matrices are calculated, their performance for the detection of stars in a sky image can be evaluated with the ”Check calibration” menu. For this purpose, one star must be chosen, for example Alioth which has not be used in the calibration process, as shown in Fig. 8. ~~ORION is run~~ after the ”Check” button is pressed ~~ar~~ analyzes each single image from the set in the image path. The calibration matrices point out that the center of the chosen star should be in a certain pixel (marked in red circle in Fig. 8). ORION also looks for the brightest pixel in a box centred in the mentioned pixel. This box has the size of the box used in the ”Default” option of ”Automatic” mode (see 2.2). The brightest pixel (marked as green circle; see Fig. 8) is assumed as the real position of the star center in the analyzed sky image.

Fig 8. ORION screenshot for checking the calibration feature using the star Alioth.

Hence, the software calculates the real star position (brightest pixel) and the one predicted by calibration matrices for all images, and then uses both positions to quantify the agreement between the predicted star position by calibration and the real one. Five panels with different analyses are provided for this verification, see Fig. 9.

Fig 9. Performance of the obtained calibrations matrices for Alioth star positions: a) azimuth obtained with the calibration for the located star position (brightest point) as a function of the real star azimuth; b) zenith obtained with the calibration for the located star position (brightest point) as a function of the real star zenith; c) absolute pixel distance between the star position given by the calibration matrices and the position of the assumed real center of the star in the image (brightest point in a square box defined by the height or width of the sky image) as a function of star azimuth angle (red dotted line represents the mean absolute distance); d) absolute pixel distance between the star position given by the calibration matrices and the position of the assumed real center of the star in the image (brightest point in a square box defined by the height or width of the sky image) as a function of star zenith angle (red dotted line represents the mean absolute distance); e) absolute pixel distance between the star position given by the calibration matrices and the position of the assumed real center of the star in the image (brightest point in a square box defined by the height or width of the sky image) for each available used image (red dotted line represents the mean absolute distance).

Figure 9a, which is the default panel shown by ORION (see Fig. 8), represents the pixel distance between the predicted and real star positions for each image in the analyzed set. This distance is between 0 and 15 arcmin in the analyzed example, being the mean distance about 7.25 arcmin (dotted red line). This means that the obtained calibration matrices predicted the center of the star Alioth with an average difference of 7.25 arcmin (it corresponds ~~in this camera~~ about 1.5 pixels) [illegible] the real position. These difference values given by the distance between predicted and real positions are also presented as a function of the star azimuth (Figs. 9b) and zenith (Fig. 9c) angles. No azimuth or zenith angle dependence is observed in the analyzed example. Finally, ORION shows the azimuth (9d) and zenith (Fig. 9e) angles, given by the calibration matrices, of the real star positions in the image (brightest pixel) as a function of the real ones angles for all analyzed sky images. As it can be observed in the example, the angles assigned by the calibration matrices to the star position in the image (brightest pixel) highly correlate with the real coordinates of the star.

This calibration check has been also carried out for 9 additional stars, see Table 1. It is observed that the mean for all stars is between 4 and 12 arcmin. The best results are obtained by Kochab with a mean of 4.50 arcmin and a standard deviation of 3.24 arcmin. The highest maximum values are observed in Dubhe, Mirach and Sirrah, which corresponds to the presence of hot pixels in positions close to the stars that are not well identified. In general, the mean accuracy for the 10 stars is about 9.0 arcmin (1.7 pixels) and the mean precision, given by the standard deviation, is about 7.5 arcmin (1.4 pixels).

4 Conclusions

This paper presents ORION, a new software application, which provides the geometrical calibration of all-sky cameras using a sky image set under cloudless conditions. An example of use has shown the capability of this application to obtain the azimuth and zenith angles viewed by each pixel of an all-sky camera. The accuracy of the calibration depends on the chosen stars and the sky positions scanned by them. This accuracy can be also checked with ORION itself. A simple calibration was able to estimate the star positions with an average accuracy around 9.0 arcmin ~~on average~~ in the observed example with a camera with 5.4 arcmin/pixel resolution (1.7 pixels). The averaged precision in this case is about 7.5 arcmin (1.4 pixels). We encourage other users and

Table 1. Calibration check for others stars.

Star	N	Min (arcmin)	Max (arcmin)	Mean (arcmin)	Std (arcmin)
Algol	89	4.27	29.66	11.97	5.40
Alioth	110	0	14.74	7.25	2.87
Antares	53	0	21.17	9.05	4.76
Dubhe	110	0	119.0	10.06	16.74
Fomalhaut	59	0	30.66	12.0	8.05
Kochab	110	0	11.33	4.50	3.24
Mirach	110	0	74.07	10.30	10.70
Mizar	110	0	14.20	6.63	2.87
Shedar	110	0	12.37	7.54	2.75
Sirrah	110	0	121.58	11.12	17.42

Number of points (N), minimum (Min), maximum (max), mean (Mean) and standard deviation (Std) of the calibration check for different stars.

researchers to use the ORION application for easy geometrical calibration of all-sky cameras, which will be helpful to locate any body (stars, planets, Sun, Moon..[illegible] their sky images if the sky coordinates of that body are known.

Acknowledgments

This research was funded by the Ministerio de Ciencia, Innovación y Universidades (grant no. RTI2018-097864-B-I00) and by Junta de Castilla y León (grant no. VA227P20). The authors gratefully thank AERONET for the aerosol products used. Finally, the authors thank the GOA-UVa staff members R. Carracedo, D. González-Fernández, S. Herrero and P. Martín, who helped with the maintenance of the used camera.

References

1. McGuffie K, Henderson-Sellers A. Almost a century of “imaging” clouds over the whole-sky dome. Bulletin of the American Meteorological Society. 1989;70(10):1243–1253.

2. Tapakis R, Charalambides AG. Equipment and methodologies for cloud detection and classification: A review. Solar Energy. 2013;95:392–430. doi:https://doi.org/10.1016/j.solener.2012.11.015.

3. Cazorla A, Olmo F, Alados-Arboledas L. Development of a sky imager for cloud cover assessment. JOSA A. 2008;25(1):29–39.

4. Wacker S, Groebner J, Zysset C, Diener L, Tzoumanikas P, Kazantzidis A, et al. Cloud observations in Switzerland using hemispherical sky cameras. Journal of Geophysical Research: Atmospheres. 2015;120(2):695–707.

5. Calbo J, Sabburg J. Feature extraction from whole-sky ground-based images for cloud-type recognition. Journal of Atmospheric and Oceanic Technology. 2008;25(1):3–14.

6. Long CN, Sabburg JM, Calbó J, Pagès D. Retrieving cloud characteristics from ground-based daytime color all-sky images. Journal of Atmospheric and Oceanic Technology. 2006;23(5):633–652.

7. Ghonima MS, Urquhart B, Chow CW, Shields JE, Cazorla A, Kleissl J. A method for cloud detection and opacity classification based on ground based sky imagery. Atmospheric Measurement Techniques. 2012;5(11):2881–2892. doi:10.5194/amt-5-2881-2012.

8. Yabuki M, Shiobara M, Nishinaka K, Kuji M. Development of a cloud detection method from whole-sky color images. Polar Science. 2014;8(4):315–326.

9. Liu S, Zhang L, Zhang Z, Wang C, Xiao B. Automatic cloud detection for all-sky images using superpixel segmentation. IEEE Geoscience and Remote Sensing Letters. 2014;12(2):354–358.

10. Koehler T, Johnson R, Shields J. Status of the whole sky imager database. Proc Cloud Impacts on DOD Operations and Systems, El Segundo, CA, USA, Department of Defense. 1991; p. 77–80.

11. Janeiro FM, Carretas F, Kandler K, Ramos PM, Wagner F. Automated cloud base height and wind speed measurement using consumer digital cameras. In: Proc. IMEKO World Congress; 2012.

12. Savoy FM, Dev S, Lee YH, Winkler S. Stereoscopic cloud base reconstruction using high-resolution whole sky imagers. In: 2017 IEEE International Conference on Image Processing (ICIP). IEEE; 2017. p. 141–145.

13. Wang Q, Liang J, Hu ZJ, Hu HH, Zhao H, Hu HQ, et al. Spatial texture based automatic classification of dayside aurora in all-sky images. Journal of Atmospheric and Solar-Terrestrial Physics. 2010;72(5):498–508. doi:https://doi.org/10.1016/j.jastp.2010.01.011.

14. Kenyon DA, Watson WT. The All Sky Camera Fireball Detector. In: Society for Astronomical Sciences Annual Symposium. vol. 24; 2005. p. 11.

15. Trigo-Rodriguez JM, Madiedo JM, Gural PS, Castro-Tirado AJ, Llorca J, Fabregat J, et al. Determination of meteoroid orbits and spatial fluxes by using high-resolution all-sky CCD cameras. In: Advances in Meteoroid and Meteor Science. Springer; 2008. p. 231–240.

16. Zibordi G, Voss KJ. Geometrical and spectral distribution of sky radiance: comparison between simulations and field measurements. Remote Sensing of Environment. 1989;27(3):343–358.

17. Román R, Antón M, Cazorla A, de Miguel A, Olmo FJ, Bilbao J, et al. Calibration of an all-sky camera for obtaining sky radiance at three wavelengths. Atmospheric Measurement Techniques. 2012;5(8):2013–2024. doi:10.5194/amt-5-2013-2012.

18. Antuña Sánchez JC, Román R, Cachorro VE, Toledano C, López C, González R, et al. Relative sky radiance from multi-exposure all-sky camera images. Atmospheric Measurement Techniques. 2021;14(3):2201–2217. doi:10.5194/amt-14-2201-2021.

19. Calbó J, Pages D, González JA. Empirical studies of cloud effects on UV radiation: A review. Reviews of Geophysics. 2005;43(2).

20. Antón M, Gil J, Cazorla A, Fernández-Gálvez J, Foyo-Moreno I, Olmo F, et al. Short-term variability of experimental ultraviolet and total solar irradiance in Southeastern Spain. Atmospheric environment. 2011;45(28):4815–4821.

21. Kreuter A, Emde C, Blumthaler M. Measuring the influence of aerosols and albedo on sky polarization. Atmospheric Research. 2010;98(2):363–367. doi:https://doi.org/10.1016/j.atmosres.2010.07.010.

22. Zhang W, Cao Y, Zhang X, Yang Y, Ning Y. Angle of sky light polarization derived from digital images of the sky under various conditions. Appl Opt. 2017;56(3):587–595. doi:10.1364/AO.56.000587.

23. Cazorla A, Olmo FJ, Alados-Arboledas L. Using a Sky Imager for aerosol characterization. Atmospheric Environment. 2008;42(11):2739–2745. doi:https://doi.org/10.1016/j.atmosenv.2007.06.016.

24. Kreuter A, Blumthaler M. Feasibility of polarized all-sky imaging for aerosol characterization. Atmospheric Measurement Techniques. 2013;6(7):1845–1854.

25. Román R, Antuña Sánchez JC, Cachorro VE, Toledano C, Torres B, Mateos D, et al. Retrieval of aerosol properties using relative radiance measurements from an all-sky camera. Atmospheric Measurement Techniques Discussions. 2021;2021:1–69. doi:10.5194/amt-2021-204.

26. Martínez-Chico M, Batlles F, Bosch J. Cloud classification in a mediterranean location using radiation data and sky images. Energy. 2011;36(7):4055–4062.

27. Román R, Cazorla A, Toledano C, Olmo FJ, Cachorro VE, de Frutos A, et al. Cloud cover detection combining high dynamic range sky images and ceilometer measurements. Atmospheric Research. 2017;196:224–236. doi:https://doi.org/10.1016/j.atmosres.2017.06.006.

28. Román R, Torres B, Fuertes D, Cachorro VE, Dubovik O, Toledano C, et al. Remote sensing of lunar aureole with a sky camera: Adding information in the nocturnal retrieval of aerosol properties with GRASP code. Remote Sensing of Environment. 2017;196:238–252. doi:https://doi.org/10.1016/j.rse.2017.05.013.

29. Alonso-Montesinos J, Batlles FJ, Portillo C. Solar irradiance forecasting at one-minute intervals for different sky conditions using sky camera images. Energy Conversion and Management. 2015;105:1166–1177. doi:https://doi.org/10.1016/j.enconman.2015.09.001.

30. Kazantzidis A, Tzoumanikas P, Blanc P, Massip P, Wilbert S, Ramirez-Santigosa L. Short-term forecasting based on all-sky cameras. In: Renewable energy forecasting. Elsevier; 2017. p. 153–178.

31. Kataoka R, Miyoshi Y, Shigematsu K, Hampton D, Mori Y, Kubo T, et al. Stereoscopic determination of all-sky altitude map of aurora using two ground-based Nikon DSLR cameras. Annales Geophysicae. 2013;31(9):1543–1548. doi:10.5194/angeo-31-1543-2013.

32. Nguyen DA, Kleissl J. Stereographic methods for cloud base height determination using two sky imagers. Solar Energy. 2014;107:495–509.

33. Scaramuzza D, Martinelli A, Siegwart R. A toolbox for easily calibrating omnidirectional cameras. In: 2006 IEEE/RSJ International Conference on Intelligent Robots and Systems. IEEE; 2006. p. 5695–5701.

34. Crispel P, Roberts G. All-sky photogrammetry techniques to georeference a cloud field. Atmospheric measurement techniques. 2018;11(1):593–609.

35. Lalonde JF, Narasimhan SG, Efros AA. What do the sun and the sky tell us about the camera? International Journal of Computer Vision. 2010;88(1):24–51.

36. Urquhart B, Kurtz B, Kleissl J. Sky camera geometric calibration using solar observations. Atmospheric Measurement Techniques. 2016;9(9):4279–4294.

37. Mori Y, Yamashita A, Tanaka M, Kataoka R, Miyoshi Y, Kaneko T, et al. Calibration of fish-eye stereo camera for aurora observation. In: Proceedings of the International Workshop on Advanced Image Technology (IWAIT2013); 2013. p. 729–734.

38. Barghini D, Gardiol D, Carbognani A, Mancuso S. Astrometric calibration for all-sky cameras revisited. Astronomy & Astrophysics. 2019;626:A105.

39. Bennouna YS, Cachorro VE, Torres B, Toledano C, Berjón A, de Frutos AM, et al. Atmospheric turbidity determined by the annual cycle of the aerosol optical depth over north-center Spain from ground (AERONET) and satellite (MODIS). Atmospheric Environment. 2013;67:352 – 364. doi:https://doi.org/10.1016/j.atmosenv.2012.10.065.

40. Román R, Bilbao J, de Miguel A. Uncertainty and variability in satellite-based water vapor column, aerosol optical depth and Angström exponent, and its effect on radiative transfer simulations in the Iberian Peninsula. Atmospheric Environment. 2014;89:556 – 569. doi:https://doi.org/10.1016/j.atmosenv.2014.02.027.

41. Cachorro VE, Burgos MA, Mateos D, Toledano C, Bennouna Y, Torres B, et al. Inventory of African desert dust events in the north-central Iberian Peninsula in 2003–2014 based on sun-photometer–AERONET and particulate-mass–EMEP data. Atmospheric Chemistry and Physics. 2016;16(13):8227–8248. doi:10.5194/acp-16-8227-2016.

42. Rhodes BC. PyEphem: astronomical ephemeris for Python. Astrophysics Source Code Library. 2011; p. ascl–1112.

43. OpenCV. OpenCV: Operations on arrays;. Available from: `https://docs.opencv.org/3.4/d2/de8/group__core__array.html#gab473bf2eb6d14ff97e89b355dac20707`.

44. Zotti G, Hoffmann SM, Wolf A, Chéreau F, Chéreau G. The Simulated Sky: Stellarium for Cultural Astronomy Research. Journal of Skyscape Archaeology. 2020;6(2):221–258–221–258. doi:10.1558/jsa.17822.

45. Brummelen GV. Heavenly Mathematics: The Forgotten Art of Spherical Trigonometry. Princeton University Press; 2013.

Click here to access/download;Figure;Figure01.tif

Figure01.png

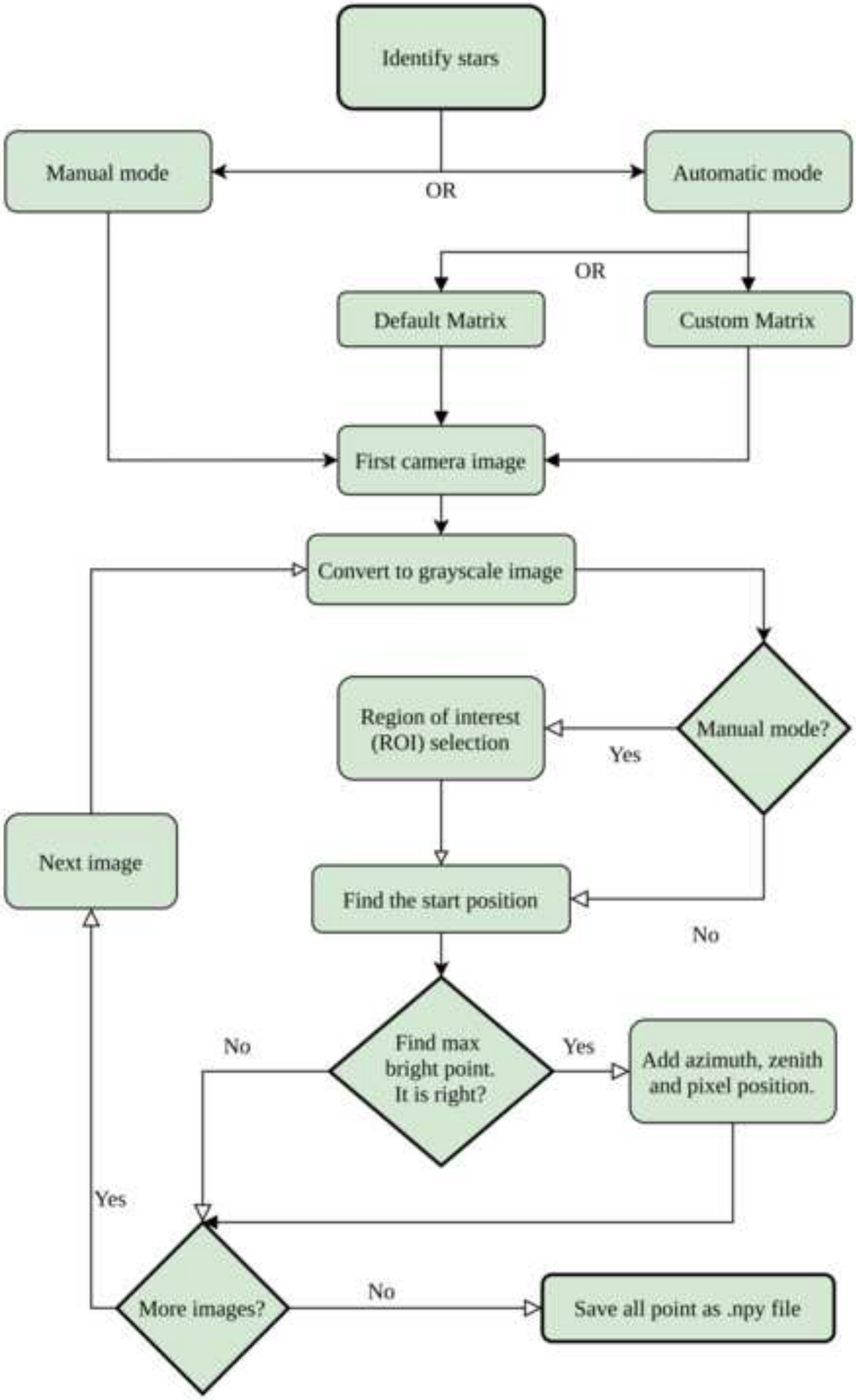

Figure02

Click here to access/download;Figure;Figure02.tif

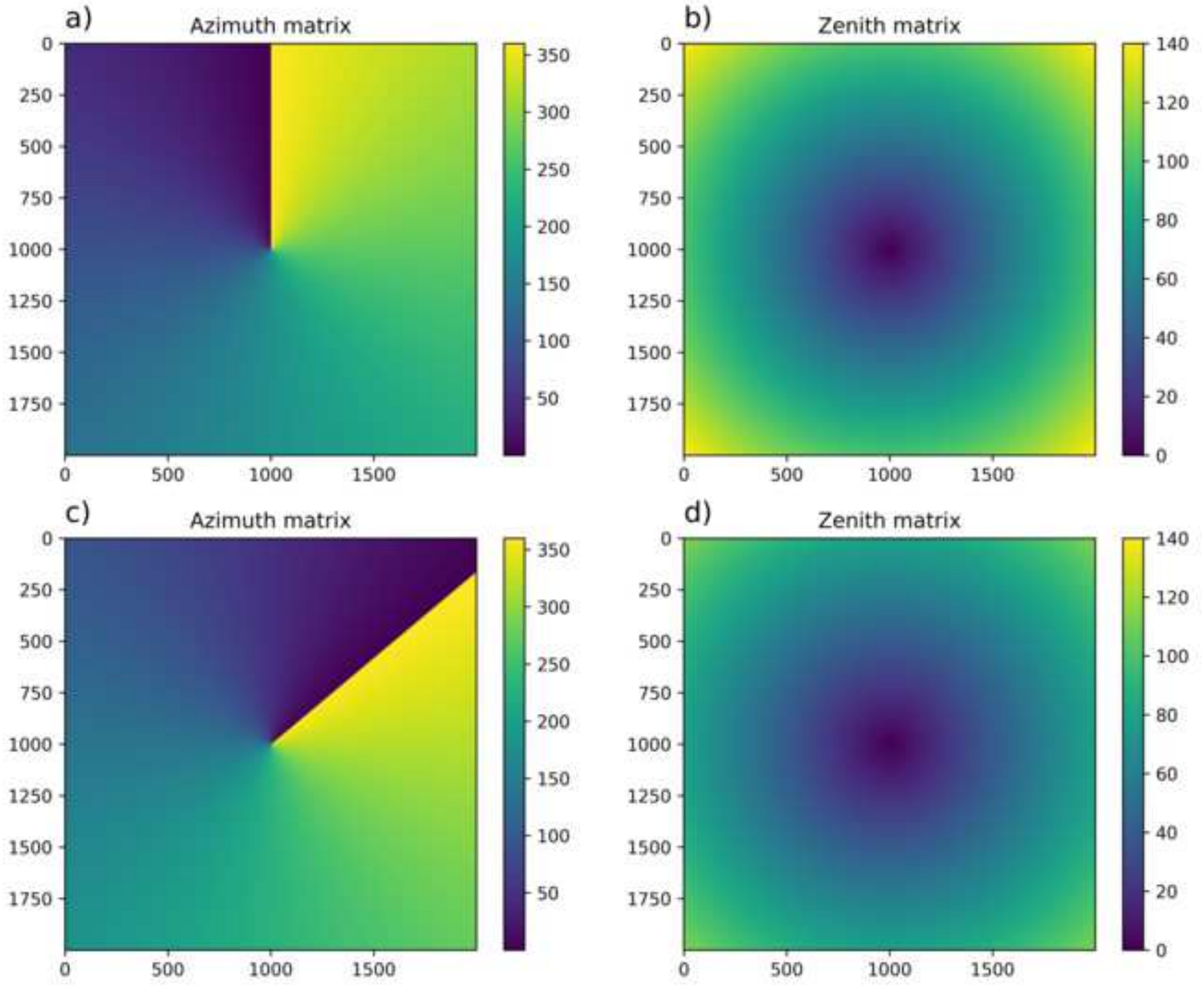

Figure02.png

Figure03

Click here to access/download;Figure;Figure03.tif

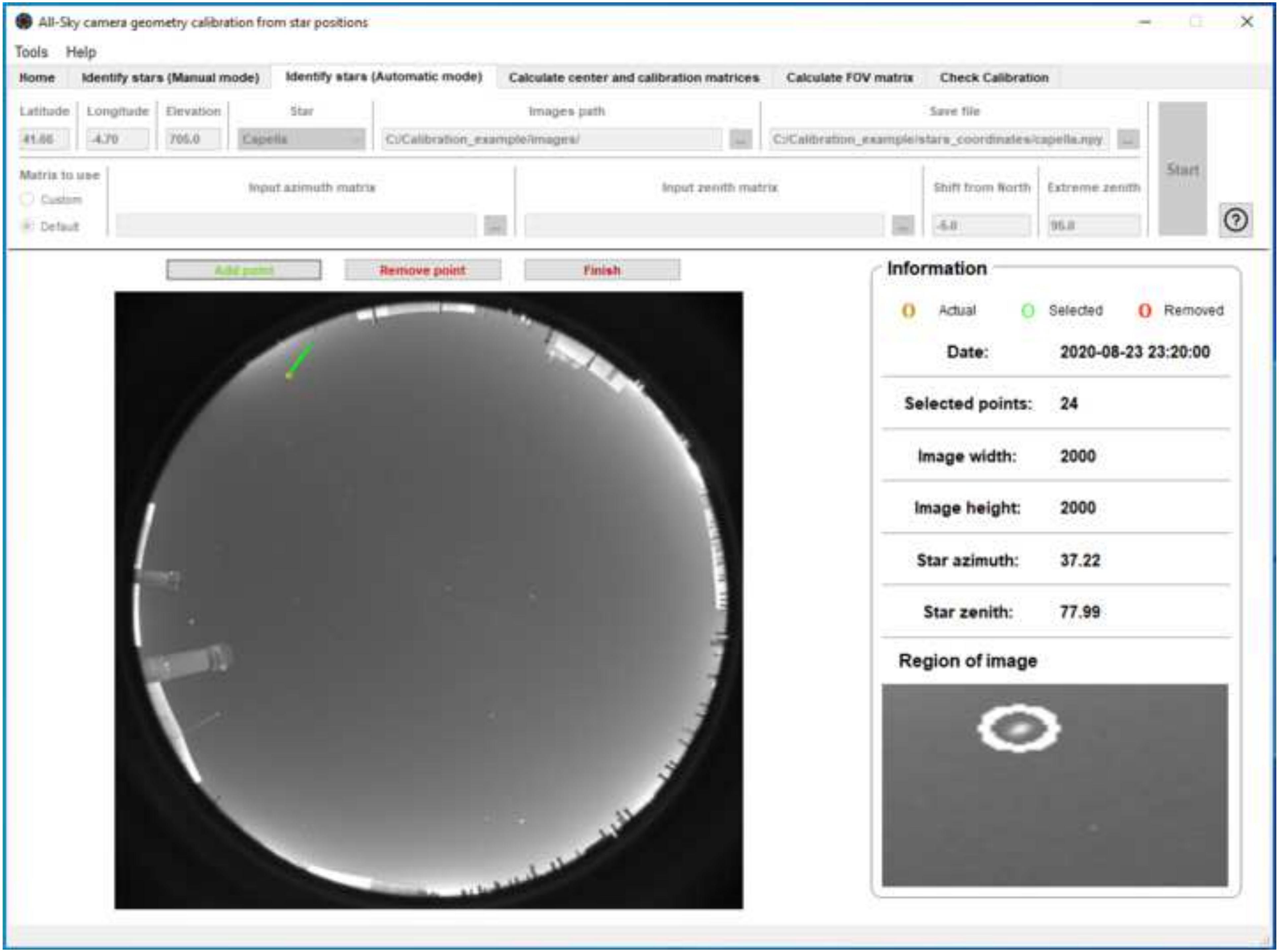

Figure03.png

Figure04

Click here to access/download;Figure;Figure04.tif

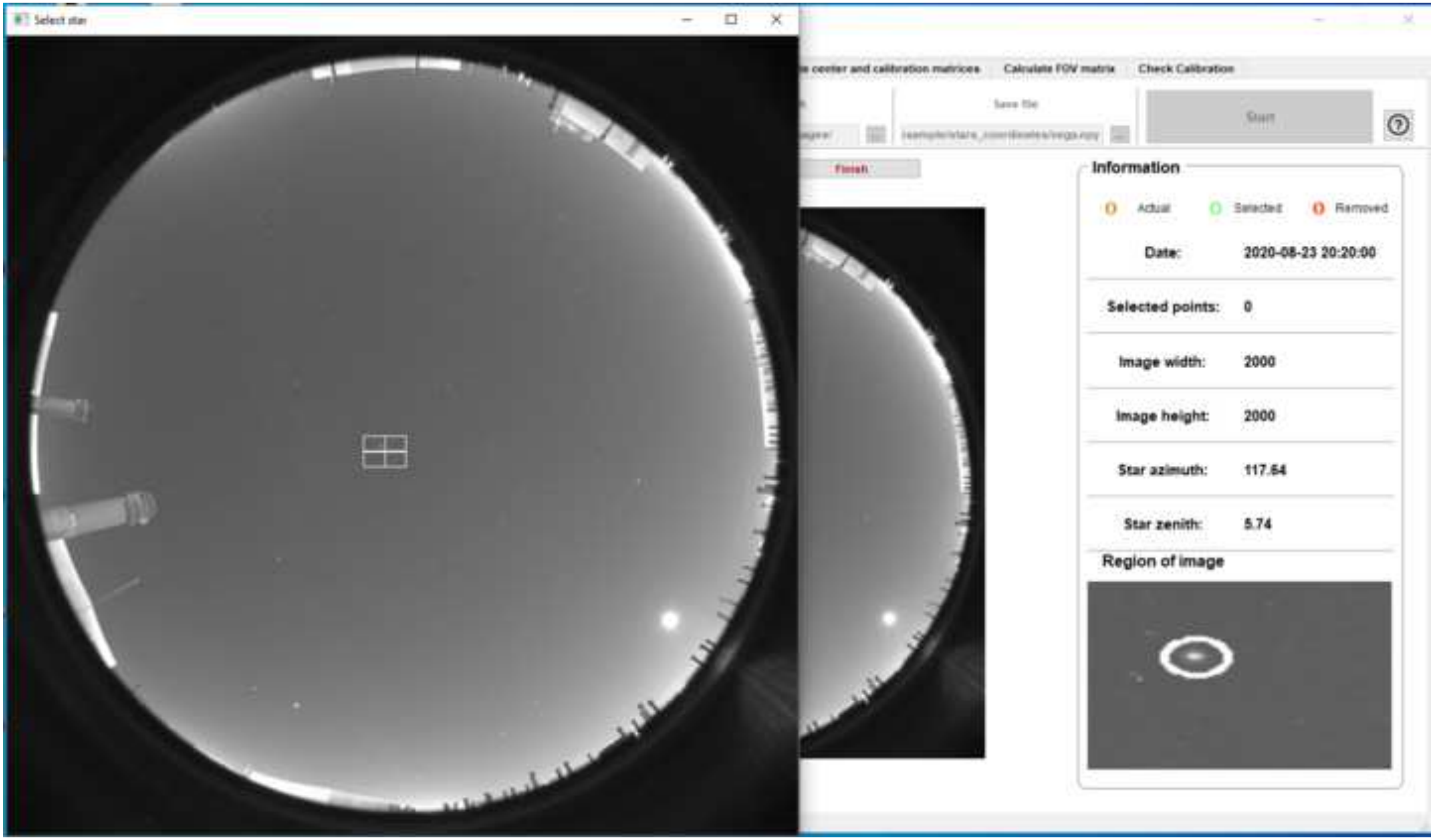

Figure04.png

Figure05

Click here to access/download;Figure;Figure05.tif

Figure05.png

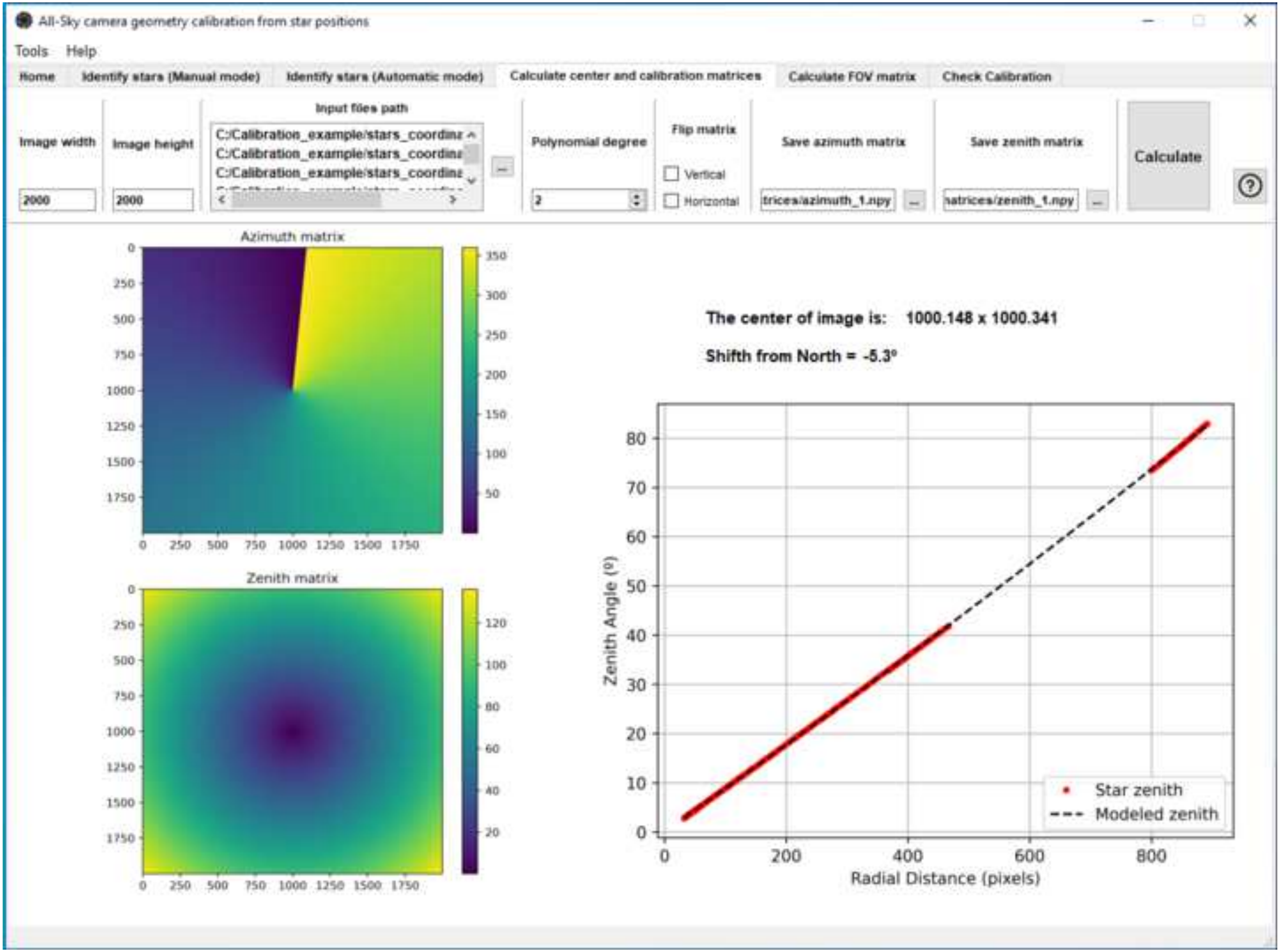

Figure06

Click here to access/download;Figure;Figure06.tif

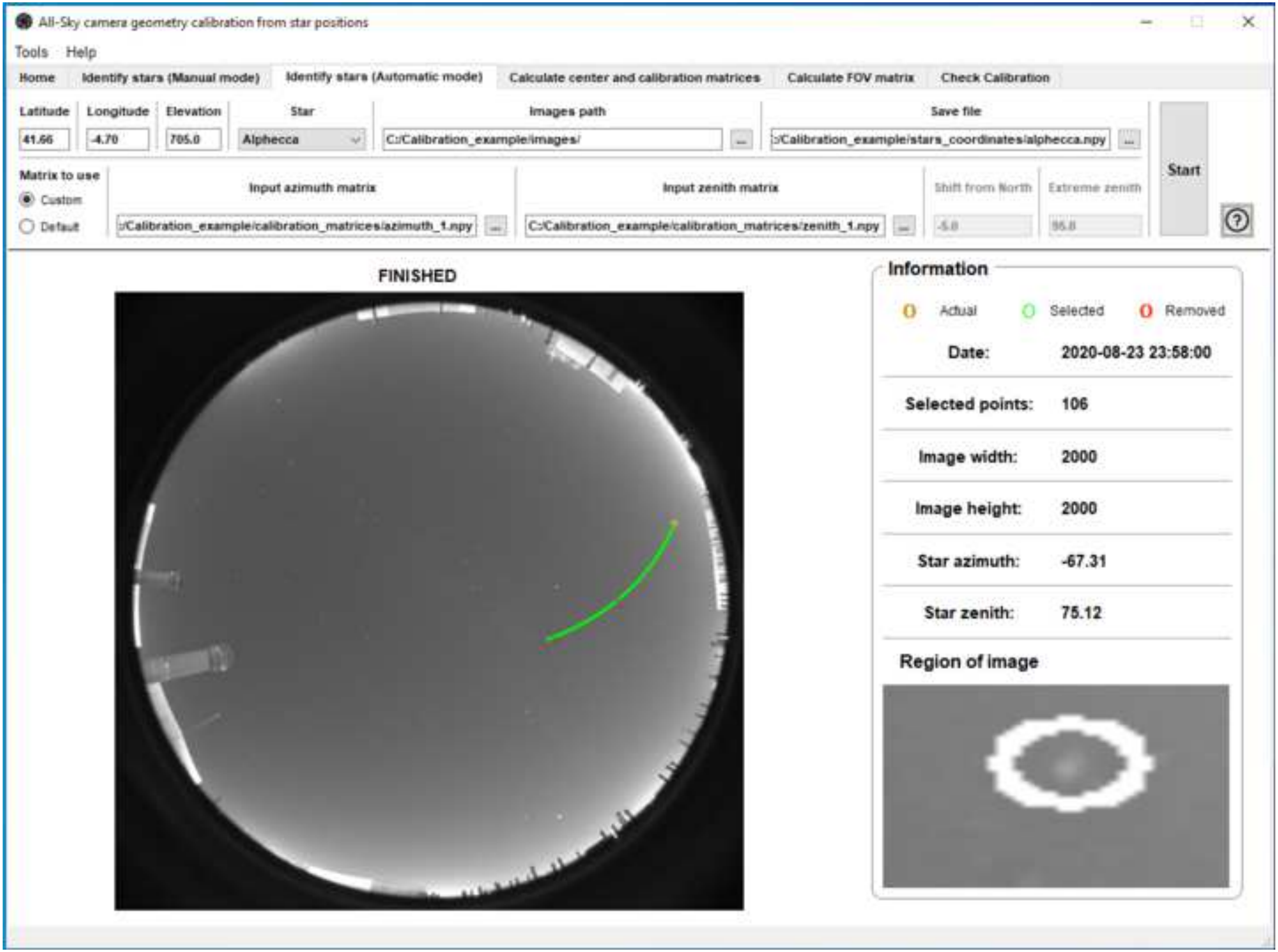

Figure06.png

Figure07 Click here to access/download;Figure;Figure07.tif

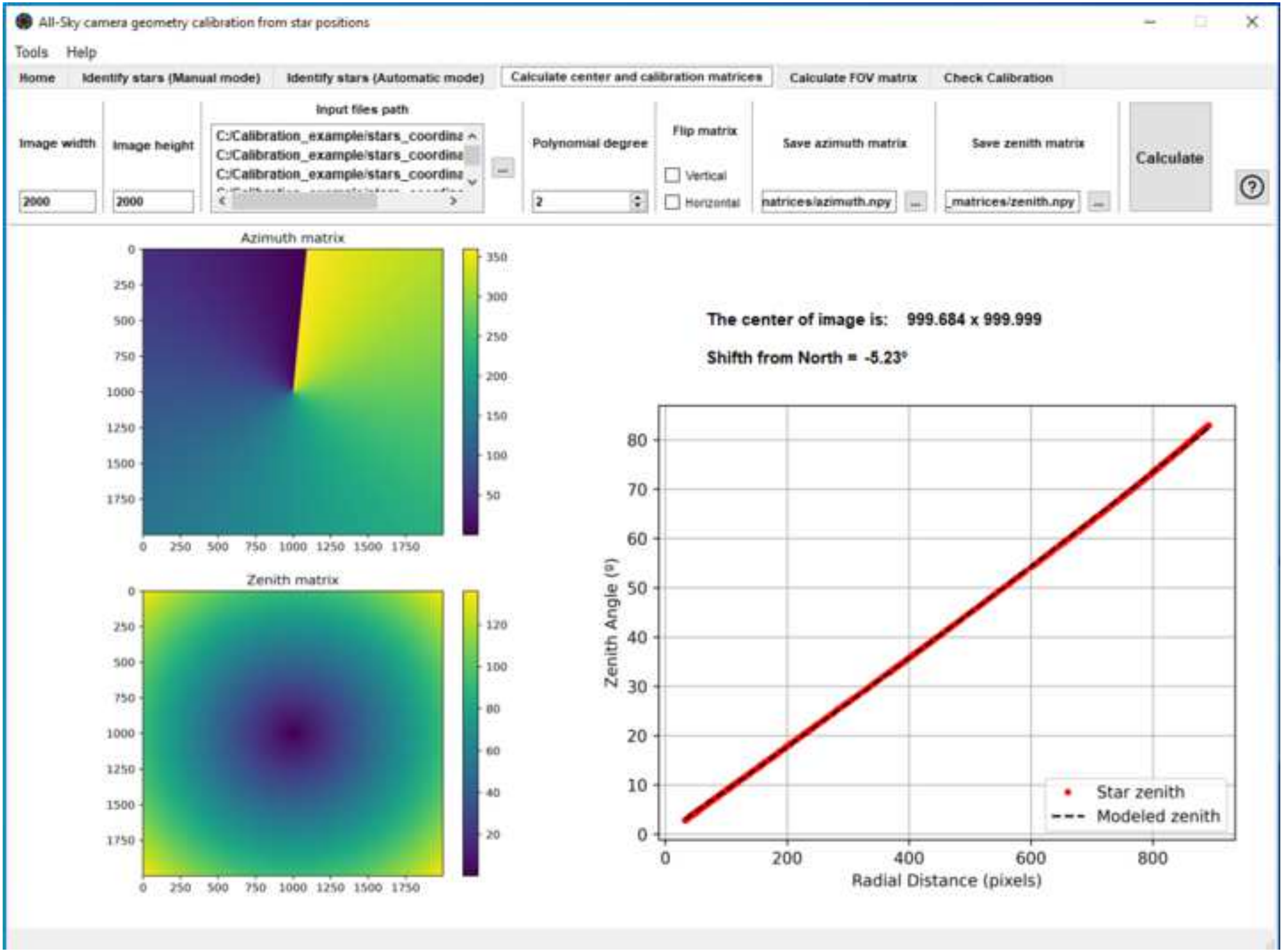

Figure07.png

Figure08

Click here to access/download;Figure;Figure08.tif

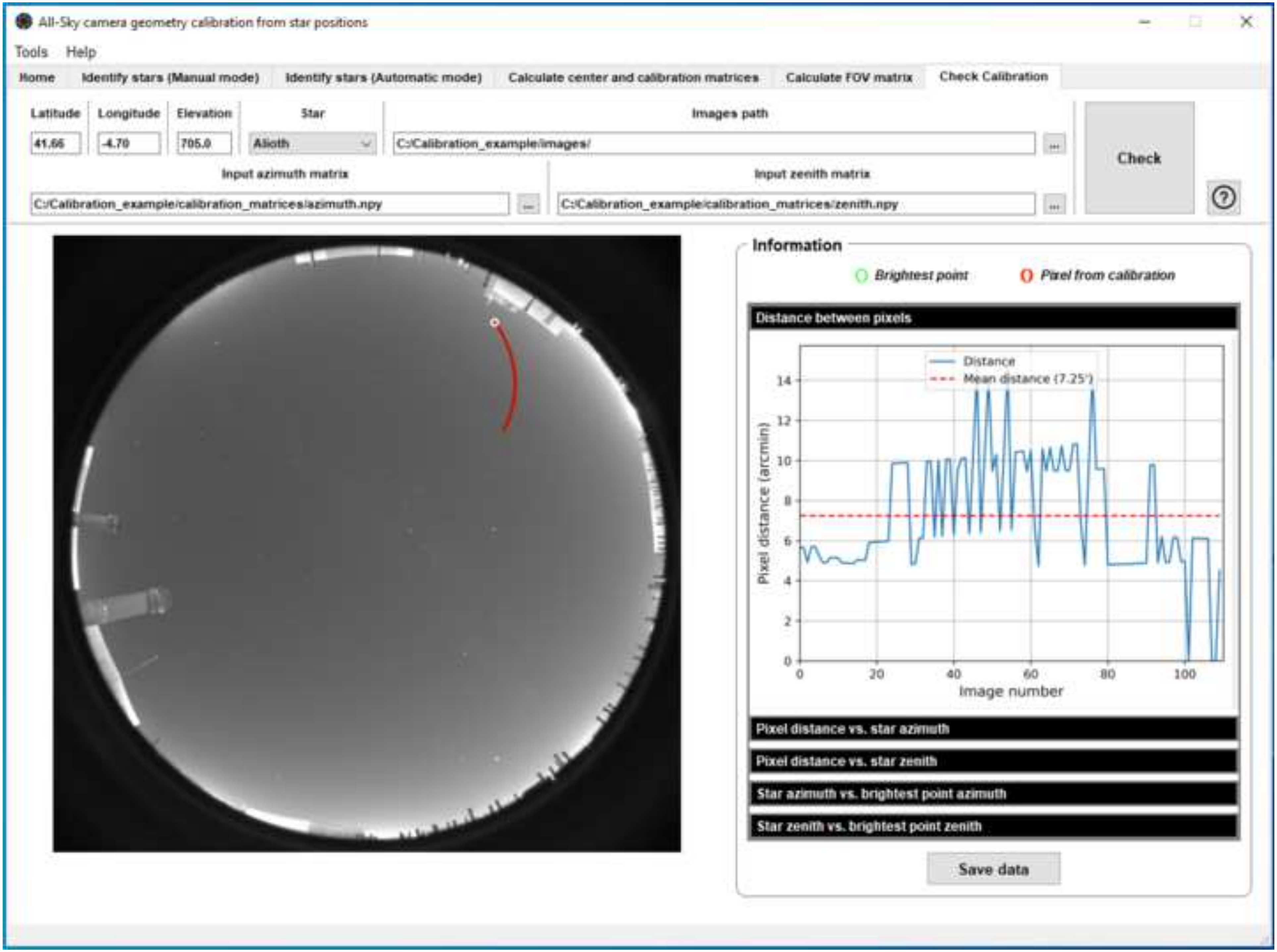

Figure08.png

Figure09

Click here to access/download;Figure;Figure09.tif

Figure09.png

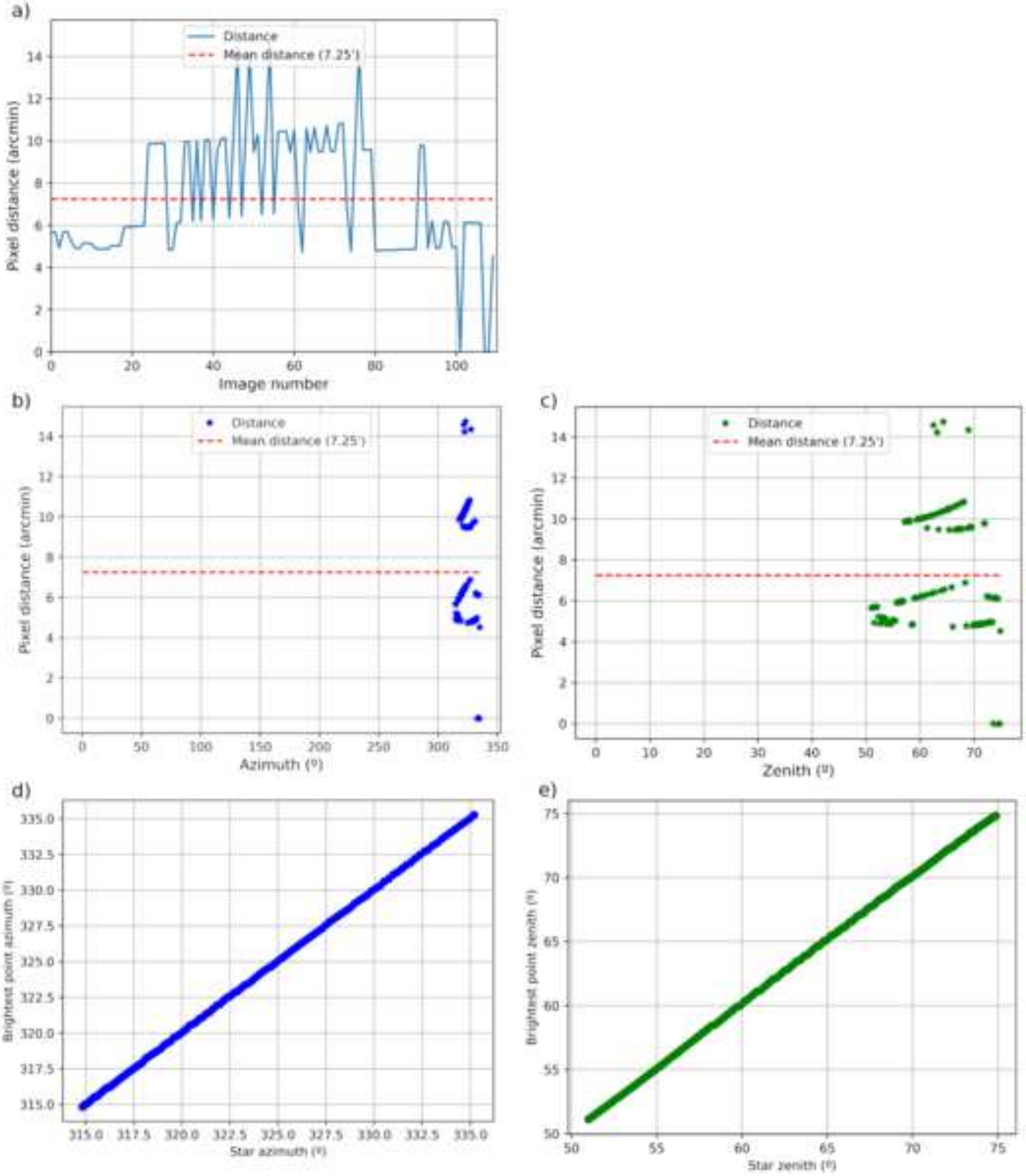

Supporting Information 01

Click here to access/download
Supporting Information
Suplementary_01.tif
