# Supplementary material for: ORION software tool for the geometrical calibration of all-sky cameras
Source: PLoS One. 2022 Mar 31;17(3):e0265959. doi: 10.1371/journal.pone.0265959 (PMC8970509; doi:10.1371/journal.pone.0265959)
Supplement: Attachment — Submitted filename: Reviewer_2_ct_RR.pdf [file pone.0265959.s003.pdf]

Response to the Reviewer #2 comments for the manuscript “ORION software tool for the geometrical calibration of all-sky cameras” By Juan Carlos Antuña Sánchez et al. in PLOS ONE

First, we are grateful for the effort of Referee #2 and her/his review in detail. Reviewer comments are in black font (RC), and author comments (AC) in red font.

Reviewer #2:

RC: This paper developed a new open-source software, ORION, to conduct geometrical calibration to all-sky cameras under restricted conditions (e.g., cloudless). From the writing perspective, it is good to provide the principles underlying the software and an example to show how it works. However, I think there are too many operation-level description (e.g., what button to click in this software), which makes some part of the paper more like a user guide. It would be improved if the authors deliver it from a perspective of an academic study instead of from a user guide scope (see detail in section-wise comments). A few issues about formats (e.g., thousand separator) and grammar need to be double checked (see detail in line-wise comments).

Section-wise comments:

RC: Section 2.4 Too detail in terms of the techniques (e.g., different forms of output). It makes it more like a user guide not academic study.

AC: This section has been removed, as it is descriptive of additional utilities of the application.

RC: Section 3 Some operations may not be needed because that is more like a user-guide. For example, you mentioned what will happen after you click a specific button (e.g., “Start” and “Check” buttons).

AC: This kind of statements has been corrected in the new manuscript.

RC: Figure 1 You may want to include a legend for different shapes in your flow chart.

AC: The arrowheads in the flowchart have been fixed. In the description of Figure 1 the meaning of the rhombuses has been added with the following sentence "The rhombuses represent decisions made by the user."

RC: Figures 3-9 I do not suggest using screenshots of the software to deliver the functions because that makes it like a user guide. You may simply separate different functions or different stages of processing to different sections in your paper and simply visualize the results of each stage (or functions) you get, like Figure 2.

AC: Figure 4 has been removed and Figures 5, 6, 7 have been substituted to improve the visualization of the results following the reviewer comment.

Line-wise comments
RC: L47 python3->Python 3

AC: It has been changed.

RC: L47 please give a reference link to Qt5. I am not sure what it is.

AC: We have added a reference.

RC: L59 This is the second time you mention GOA-UVa but your first time mentions the full name. You may want to mention it in line 51

AC: It has been done.

RC: L67 3096 X 2080 -> 3,096 X 2,080. You may need thousand separators.

AC: It has been corrected.

RC: L77 a space before “To”?

AC: A space has been added.

RC: L157 You may want no indent before “where”

AC: It has been corrected.

RC: L183 10,201thousand separators
RC: L184 10,201thousand separators
RC: L190 10,201thousand separators

AC: All thousands separators have been included.

RC: L338 difference -> different?

AC: Done.
